# Severe ocular complications of SJS/TEN and associations among pre-onset, acute, and chronic factors: a report from the international ophthalmology collaborative group

**DOI:** 10.3389/fmed.2023.1189140

**Published:** 2023-06-22

**Authors:** Mayumi Ueta, Chikara Inoue, Mitsuko Nakata, Chie Sotozono, Mee Kum Kim, Tais Wakamatsu, Passara Jongkhajornpong, Hajirah Saeed, Saaeha Rauz, David Hui-Kang Ma, Kyung Chul Yoon, Vilavun Puangsricharern, Charles Bouchard, Sajjad Ahmad, Kyoung Yul Seo, Choun-Ki Joo, Jose Alvaro Pereira Gomes, James Chodosh, Shigeru Kinoshita, Satoshi Teramukai

**Affiliations:** ^1^Department of Ophthalmology, Kyoto Prefectural University of Medicine, Kyoto, Japan; ^2^Department of Biostatistics, Graduate School of Medical Science, Kyoto Prefectural University of Medicine, Kyoto, Japan; ^3^Department of Ophthalmology, Seoul National University College of Medicine, Seoul, Republic of Korea; ^4^Department of Ophthalmology, Federal University of São Paulo, São Paulo, Brazil; ^5^Department of Ophthalmology, Faculty of Medicine, Ramathibodi Hospital, Mahidol University, Bangkok, Thailand; ^6^Department of Ophthalmology, Massachusetts Eye and Ear Infirmary and Harvard Medical School, Boston, MA, United States; ^7^Birmingham & Midland Eye Centre, University of Birmingham, Birmingham, United Kingdom; ^8^Department of Ophthalmology, Chang Gung Memorial Hospital, Linkou, Taiwan; ^9^Department of Ophthalmology, Chonnam National University Medical School and Hospital, Gwangju, Republic of Korea; ^10^Department of Ophthalmology, King Chulalongkorn Memorial Hospital, Faculty of Medicine, Chulalongkorn University, Bangkok, Thailand; ^11^Department of Ophthalmology, Loyola University Health System, Chicago, IL, United States; ^12^Moorfields Eye Hospital, Institute of Ophthalmology, London, United Kingdom; ^13^Department of Ophthalmology, Severance Hospital, Institute of Vision Research, Yonsei University College of Medicine, Seoul, Republic of Korea; ^14^CK St. Mary's Eye Clinic, Seoul, Republic of Korea

**Keywords:** Stevens-Johnson syndrome (SJS), toxic epidermal necrolysis (TEN), severe ocular complications (SOC), ocular sequelae, cold medicine, common cold symptoms, onychopathy, international research collaboration

## Abstract

We formed an international research collaboration that included Japan, South Korea, Brazil, Thailand, Taiwan, the UK, and the US (682 patients from 13 hospitals between 2005 and 2020), to better evaluate the role of race, ethnicity, and other risk factors in the pathophysiology of Stevens–Johnson syndrome (SJS) and toxic epidermal necrolysis (TEN). Ophthalmologists often see SJS/TEN patients with severe ocular complications (SOC; frequency 50% SJS/TEN patients) when the patients are referred to them in the chronic stage after the acute stage has passed. Global data were collected using a Clinical Report Form, capturing pre-onset factors, as well as acute and chronic ocular findings. Key conclusions of this retrospective observational cohort study were as follows: (1) Ingestion of cold medications [acetaminophen and non-steroidal anti-inflammatory drugs (NSAIDs)] was significantly and positively correlated with trichiasis, symblepharon, and/or conjunctivalization of the cornea in the chronic stage; (2) common cold symptoms prior to onset of SJS/TEN were significantly and positively correlated with acute conjunctivitis and ocular surface erosions in the acute stage and with trichiasis and symblepharon and/or conjunctivalization of the cornea in the chronic stage; (3) patients with SJS/TEN who presented with SOC tended to be female; (4) patients less than 30 years of age are more likely to develop SOC in the acute and chronic stages of SJS/TEN; (5) patients with acute severe conjunctivitis with ocular surface erosion and pseudomembrane formation in the acute stage are more likely to develop ocular sequelae in the chronic stage; and (6) onychopathy in the acute stage was positively correlated with ocular sequelae in the chronic stage. Our findings show that the ingestion of cold medications, common cold symptoms prior to the onset of SJS/TEN, and a young age might strongly contribute to developing the SOC of SJS/TEN.

## Introduction

Stevens–Johnson syndrome (SJS) and toxic epidermal necrolysis (TEN) are rare, life-threatening, acute immune-mediated, predominantly adverse drug reactions that affect the skin and mucous membranes including those of the ocular, oral, pulmonary, and urogynecological tissues. Clinical features in the acute stage include fever, skin erythema, and sloughing of both skin and mucosal epithelia. In addition to dermatological findings, it has been reported that in the acute stage of SJS/TEN, about 80% of patients develop ocular involvement, including mild conjunctivitis, and approximately 50% of patients present with severe ocular complications (SOC) with severe conjunctivitis with ocular surface epithelial defects and pseudomembrane (1).

We have previously defined SOC in SJS/TEN in both the acute (severe conjunctivitis, ocular surface erosions, and pseudomembranes) and chronic (severe dry eye, trichiasis, symblepharon, and/or conjunctivalization of the cornea) stages (2–4).

Typically, dermatologists and burn care physicians diagnose and treat SJS/TEN patients in the acute stage, whereas ophthalmologists often see these patients in both the acute and chronic stages. In some countries, an SJS/TEN patient may only see an ophthalmologist in the chronic phase and may have had a long lapse in multi-system SJS/TEN care between the acute hospitalization and presentation to an ophthalmologist (5). Because the characteristic dermatological and systemic features of SJS/TEN are often resolved by the chronic phase, unless the patient can reliably explain the characteristic features of their condition, ophthalmologists in these cases may need to make the diagnosis of SJS/TEN.

Diagnosis of SJS/TEN by an ophthalmologist is based on a confirmed history of acute onset of high fever, a severe mucocutaneous disease with skin eruptions, and at least two mucosal tissues including the ocular surface, when the patients had ocular sequelae of SJS/TEN such as severe dry eye, trichiasis, symblepharon, and/or conjunctivalization of the cornea in the chronic stage (6–9).

An adverse drug reaction (ADR) in a genetically predisposed individual has been proposed as the most likely pathophysiological mechanism for SJS/TEN. For example, for carbamazepine-induced SJS/TEN, there is a strong association with the HLA-B*15:02 allele in Han Chinese patients in Taiwan (10) and in Thai patients (11). However, the HLA-A*31:01 allele is strongly associated with carbamazepine-induced SJS/TEN in Japanese patients (12) and patients of European ancestry (13). It has also been reported that not all patients with carbamazepine-induced SJS/TEN develop ocular symptoms (14). Allopurinol-induced SJS/TEN is strongly associated with HLA-B* 58:01 in Han Chinese (15), Caucasian (16), and Japanese patients (17), although allopurinol tends to induce SJS/TEN with fewer and less severe ocular symptoms (18) than in cases with other culprit drugs. In cold medicine-associated SJS/TEN with SOC, the HLA-A*02:06 allele showed a strong association in Japanese (6) and Korean (7, 19) patients, but the HLA-B*44:03 allele was found to be significantly associated with the cold medicine-associated SJS/TEN with SOC in Japanese (6), Western Brazilian (7, 20), Indian (7, 21), and Thai (22) patients.

Furthermore, we previously reported that 80% of Japanese patients with SJS/TEN with SOC were taking cold medication before disease onset (23). This was also found to be the case in 53% of Brazilian patients (20), 69% of Thai patients (22), and 50% of Taiwanese patients (24).

Because of strong genetic risk factors for the development of SJS/TEN with SOC that vary by population, an international collaborative research program among Japan, South Korea (7, 19, 25), Brazil (7, 20, 26), India (7, 21), Thailand (22, 27, 28), Taiwan (24), the UK (29), and the US was initiated to better characterize the genetic background and causative drug associated with SJS/TEN with SOC. Ophthalmologists often see patients only after the acute stage has passed, upon referral in the chronic stage. Relatively few articles have reported on the clinical features and associated risk factors in the acute stage as they relate to the sequelae in the chronic stage. In the current study, data from each country was pooled collectively in order to identify a correlation between the following: (1) clinical and demographic factors (age, gender, presumed causative drug, and common cold symptoms); (2) acute clinical findings including eye findings (severe conjunctivitis, erosion of ocular surface epithelium, pseudomembrane with conjunctivitis, and alopecia of eyelids), onychopathy, and oral mucosa erosions; and (3) chronic stage ocular findings, such as dry eyes, trichiasis, symblepharon, and conjunctivalization of the cornea.

## Methods

### Study design

This is a retrospective observational cohort study of patients with SJS/TEN.

### Setting and participants

The study population consisted of patients with SJS/TEN from 13 hospitals in seven countries (i.e., Japan, Korea, Brazil, Thailand, Taiwan, the United States, and the United Kingdom) between 2005 and 2020. SJS/TEN was diagnosed using standardized criteria including a confirmed history of acute onset of high fever, a severe mucocutaneous disease with skin eruptions, and involvement of at least two mucosal tissues including the ocular surface. All participating hospitals had an ophthalmology service delivering specialized care for SJS/TEN treatment. Our study was approved by the institutional review board of all the hospitals. Case Report Forms (CRF) for the retrospective study were made by all hospitals, which documented clinical findings from the pre-onset to the chronic stage, but some CRFs were missing some data, especially of pre-onset and acute stages because ophthalmologists usually saw the patients in their chronic stage.

We defined the day of onset of the SJS/TEN-specific skin rash as day 0, the pre-onset period from day −14 to day 0, the acute period from day 0 to 3 months, the sub-acute period from 3 months to 1 year, and the chronic period from 1 year afterward based on our previous study (1). Chronic findings were verified by the statements in the CRFs and/or photo-documentation of the external eye and anterior segment (3). Patients who did not meet the main diagnostic criteria for SJS/TEN and patients with substantial missing values were excluded.

### Factors

The potential association between specific clinical and demographic features and adverse outcomes (ocular surface complications in the acute stage and ocular surface sequelae in the chronic stage) were examined at three different disease stages: pre-onset, acute, and chronic (Table 1).

**Table 1:** The list of factors considered in this study.

Stage	Number of the factor	Factors
Pre-onset stage	6 factors	Taking cold medicines before the onset
		Taking antibiotics before the onset
		Taking Anticonvulsants before the onset
		Sex of patient
		Cold symptoms before the onset
		Age at the onset
Acute stage	6 factors	Severe conjunctivitis
		Erosion of ocular surface epithelium
		Pseudomembrane with conjunctivitis
		Alopecia of eyelids
		Onychopathy
		Vesiculobullous lesion of the oral cavity and lips
Chronic stage	3 factors	Dry eye
		Trichiasis
		Symblepharon or conjunctivalization on the cornea

Table 1 shows the factors used in the analysis.

1. Pre-onset stage factors include culprit drugs (cold medicines, antibiotics, and anticonvulsants), gender, common cold symptoms before onset, and age at onset of SJS/TEN. Cold medicines include over-the-counter and hospital-prescribed acetaminophen and NSAIDs. Medicines started after day 0 were excluded. If several medications were taken simultaneously, all applicable classifications were included in the analysis.
2. Acute stage factors include severe conjunctivitis, ocular surface epithelial erosions, pseudomembranes, eyelid alopecia, onychopathy, and vesiculobullous lesions of the oral cavity and lips. The presence of onychopathy at the acute stage was defined if the item of “nail deformation” and/or “lost nail at acute or subacute stage” (30) was checked in the CRF for the retrospective study.
3. Chronic stage factors include dry eye, trichiasis, and symblepharon or conjunctivalization of the cornea (3). The presence of trichiasis (2, 3) at the chronic stage was defined if the item of “Trichiasis” was checked on the CRF, if trichiasis was diagnosed by a photograph, or if there was a comment on treatment for trichiasis. The presence of symblepharon (2, 3) or conjunctivalization (2, 3) to the cornea at the chronic stage was defined if the item “symblepharon or conjunctivalization” was checked on the CRF or if symblepharon or conjunctival invasion was identified in a photograph.

### Statistical methods

#### Descriptive statistics

The numbers and percentages of each factor stratified by country were tallied except the age at onset, for which mean and standard deviation were calculated.

#### Odds ratios

For each pre-onset factor, the odds ratio and 95% confidence interval for the presence of each acute factor were estimated by country. For each pre-onset factor, the odds ratio and 95% confidence interval for the presence of each chronic factor were estimated by country. For each acute factor, the odds ratio and 95% confidence interval for the presence of each chronic factor were estimated by country. The age at onset was converted into a binary variable with 30 years of age as the cutoff value. For odds ratios that included 0 in any of the presence/absence categories, Woolf’s correction was performed, and 0.5 was added to all the categories to estimate the odds ratio and 95% confidence interval (31).

#### Integrated analysis

Using the estimated odds ratios for each country, we performed a country-integrated analysis using the Mantel–Haenszel method (32). Countries with no data on the relevant factors were excluded from the analysis. The Breslow–Day test was performed to test the heterogeneity among the countries, and an integrated odds ratio (IOR) was not estimated if there was significant heterogeneity among countries (value of *p* < 0.05) (32) and if the pooled sample size was below 15.

## Results

### Participant flow

**Figure 1:**
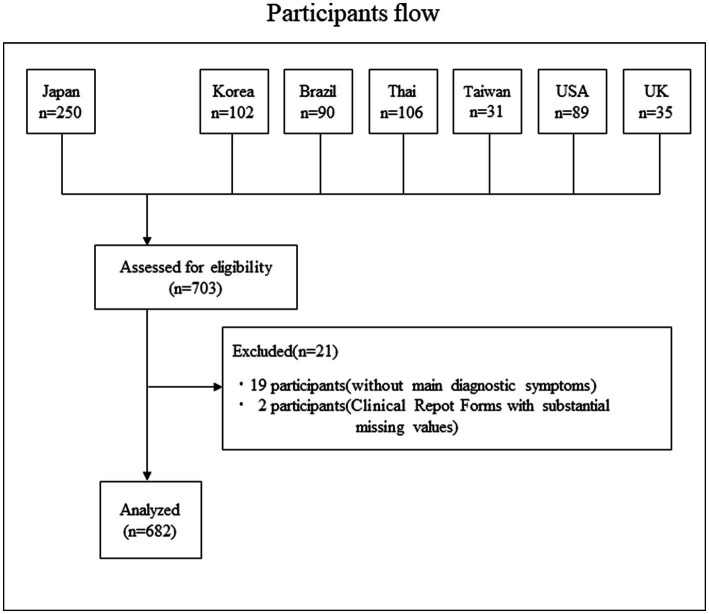
Participants flow. In total, we collected data on 703 cases; 19 patients who did not meet the main diagnostic criteria for SJS/TEN and two patients with substantial missing values were excluded, resulting in 682 participants.

The participant flow is shown in Figure 1. In total, we collected data on 703 cases; 19 patients who did not meet the main diagnostic criteria for SJS/TEN and two patients with substantial missing values were excluded, resulting in 682 participants.

### Descriptive statistics

Table 2 shows the numbers and percentages of each factor by country.

**Table 2:** Characteristics of each variables in the preset stage and outcomes of each variables in the acute and chronic stage.

(A) Pre-onset variables						
Country	Taking cold medicines	Taking antibiotics	Taking Anticonvulsants	Sex of patient (Male = 1)	Cold symptom	Age at the onset
Japan (*N* = 250)	157/190 (82.6%)	68/197 (34.5%)	16/197 (8.1%)	101/250 (40.4%)	187/241 (77.6%)	27.9 ± 19.0
South Korea (*N* = 94)	49/80 (61.3%)	20/79 (25.3%)	6/79 (7.6%)	37/94 (39.4%)	68/88 (77.3%)	37.5 ± 22.2
Brazil (*N* = 90)	53/85 (62.4%)	17/85 (20.0%)	24/85 (28.2%)	40/90 (44.4%)	63/89 (70.8%)	23.4 ± 15.9
Thai (*N* = 101)	32/68 (47.1%)	41/68 (60.3%)	5/68 (7.4%)	36/101 (35.6%)	60/92 (65.2%)	29.8 ± 18.3
Taiwan (*N* = 27)	11/22 (50.0%)	2/22 (9.1%)	4/22 (18.2%)	7/27 (25.9%)	18/27 (66.7%)	32.2 ± 19.8
USA (*N* = 88)	13/44 (29.5%)	9/44 (20.5%)	3/44 (6.8%)	28/88 (31.8%)	23/80 (28.8%)	27.7 ± 19.0
UK (*N* = 32)	2/10 (20.0%)	6/10 (60.0%)	2/10 (20.0%)	10/32 (31.3%)	18/26 (69.2%)	29.0 ± 18.8
Total (*N* = 682)	317/499 (63.5%)	163/505 (32.3%)	60/505 (11.9%)	259/682 (38.0%)	437/643 (68.0%)	29.1 ± 19.4
						※Mean ± SD.

(B) Acute stage variables						
Country	Severe conjunctivitis	Erosion of ocular surface epithelium	Pseudomembrane with conjunctivitis	Alopecia of eyelids	Onychopathy	Vesiculobullous lesion of oral cavity and lips
Japan (*N* = 250)	72/72 (100.0%)	42/47 (89.4%)	42/46 (91.3%)	16/30 (53.3%)	197/226 (87.2%)	194/195 (99.5%)
South Korea (*N* = 94)	63/77 (81.8%)	49/71 (69.0%)	N/A	49/81 (60.5%)	51/94 (54.3%)	81/82 (98.8%)
Brazil (*N* = 90)	89/89 (100.0%)	26/27 (96.3%)	N/A	64/84 (76.2%)	86/90 (95.6%)	88/88 (100.0%)
Thai (*N* = 101)	79/85 (92.9%)	72/77 (93.5%)	67/70 (95.7%)	51/85 (60.0%)	68/100 (68.0%)	86/89 (96.6%)
Taiwan (*N* = 27)	21/25 (84.0%)	25/26 (96.2%)	14/19 (73.7%)	6/18 (33.3%)	23/27 (85.2%)	25/26 (96.2%)
USA (*N* = 88)	37/44 (84.1%)	40/48 (83.3%)	31/43 (72.1%)	13/37 (35.1%)	62/88 (70.5%)	57/57 (100.0%)
UK (*N* = 32)	22/23 (95.7%)	19/21 (90.5%)	9/11 (81.8%)	13/21 (61.9%)	18/27 (66.7%)	28/29 (96.6%)
Total (*N* = 682)	383/415 (92.3%)	273/317 (86.1%)	163/189 (86.2%)	212/356 (59.6%)	505/652 (77.5%)	559/566 (98.8%)

(C) Chronic stage variables			
Country	Dry eye	Trichiasis	Symblepharon or conjunctivalization to the cornea
Japan (*N* = 250)	175/199 (87.9%)	185/232 (79.7%)	202/243 (83.1%)
South Korea (*N* = 94)	N/A	45/64 (70.3%)	45/64 (70.3%)
Brazil (*N* = 90)	78/78 (100.0%)	54/78 (69.2%)	72/78 (92.3%)
Thai (*N* = 101)	92/95 (96.8%)	73/99 (73.7%)	75/99 (75.8%)
Taiwan (*N* = 27)	21/24 (87.5%)	17/24 (70.8%)	9/24 (37.5%)
USA (*N* = 88)	68/85 (80.0%)	57/83 (68.7%)	40/83 (48.2%)
UK (*N* = 32)	31/32 (96.9%)	16/31 (51.6%)	23/31 (74.2%)
Total (*N* = 682)	465/513 (90.6%)	447/611 (73.2%)	466/622 (74.9%)

### Estimated odds ratios between individual factors

#### Association between pre-onset stage factors and acute stage factors

There was a statistically significant association in the nine combinations between pre-onset factors and acute stage factors. Of these, one combination was excluded for the presence of heterogeneity by the Breslow–Day test, leaving eight combinations.

Pre-onset common cold symptoms were positively correlated with severe conjunctivitis (IOR = 3.23; 95%CI = [1.47, 7.12]) and ocular surface erosion (IOR = 3.15; 95%CI = [1.54, 6.45]) in the acute stage. Table 3 shows the results of associations between pre-onset and acute findings. The antibiotics had no statistical correlation with acute findings. Anticonvulsants had a negative correlation with severe conjunctivitis (IOR = 0.33; 95%CI = [0.12, 0.94]) and vesiculobullous lesion (IOR = 0.21; 95%CI = [0.06, 0.80]). Supplementary Table S1A shows the associations between taking anticonvulsants before the onset and findings in the acute stage. Age at onset was negatively correlated with ocular manifestations such as severe conjunctivitis (IOR = 0.42; 95%CI = [0.20, 0.91]), ocular surface erosion (IOR = 0.43; 95%CI = [0.21, 0.87]), pseudomembrane (IOR = 0.25; 95%CI = [0.09, 0.65]), and with onychopathy at onset (IOR = 0.61; 95%CI = [0.41, 0.90]). Supplementary Table S2A shows the result of the association of age and acute findings.

**Table 3:** Associations between pre-onset and acute findings.

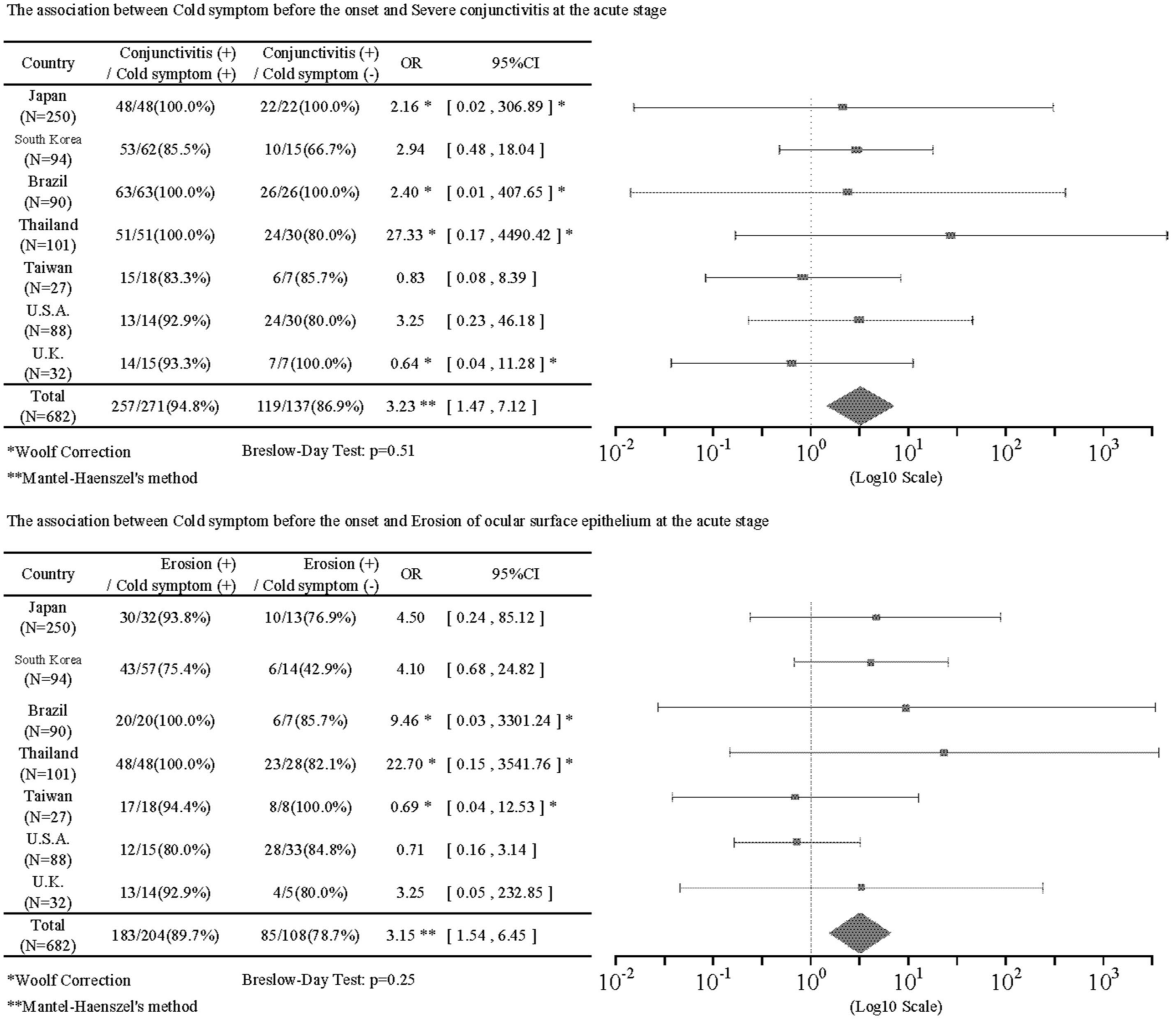

#### Associations between pre-onset stage factors and chronic stage factors

There was a statistically significant association in the seven combinations between the pre-onset factors and the chronic stage factors. The intake of cold medicine was positively correlated with trichiasis (IOR = 1.73; 95%CI = [1.08, 2.77]) and symblepharon or conjunctivalization of the cornea (IOR = 1.93; 95%CI = [1.18, 3.17]) in the chronic stage. Pre-onset common cold symptoms were positively correlated with trichiasis (IOR = 1.80; 95%CI = [1.20, 2.71]) and symblepharon or conjunctivalization of the cornea (IOR = 1.58; 95%CI = [1.02, 2.44]) in the chronic stage. Table 4 shows the results of the combination of acute findings such as the use of cold medication and pre-onset common cold symptoms and chronic findings such as trichiasis and symblepharon or conjunctivalization of the cornea. The intake of cold medicine did not correlate with dry eye (IOR = 1.49; 95%CI = [0.69, 3.25]). Common cold symptoms were also not correlated with dry eye (IOR = 1.19; 95%CI = [0.61, 2.32]). The association between antibiotics and all findings in the chronic stage did not show a statistically significant correlation.

**Table 4:** The results of the combination of cold medication and pre-onset cold symptoms.

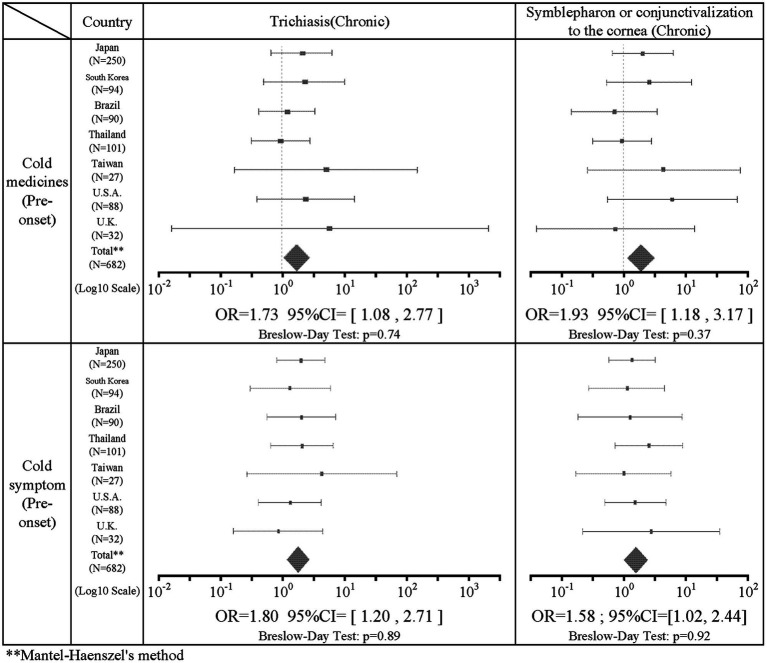

Anticonvulsants were negatively correlated with trichiasis (IOR = 0.41; 95%CI = [0.22, 0.76]). Supplementary Table S1B shows the associations between taking anticonvulsants before the onset and findings in the chronic stage. The association between age at onset and ocular findings showed a negative correlation with trichiasis (IOR = 0.58; 95%CI = [0.40, 0.84]) and symblepharon or conjunctivalization of the cornea (IOR = 0.50; 95%CI = [0.34, 0.75]). Supplementary Table S2B shows the result of the association of age and chronic findings.

#### Association between acute stage factors and chronic stage factors

There was a statistically significant association in 14 combinations between acute stage factors and chronic stage factors. Of these, two combinations related to alopecia were excluded due to the presence of heterogeneity by the Breslow–Day test. One combination for vesiculobullous lesion was excluded due to its small sample size, leaving 12 significant combinations. Between the acute and chronic stage factors, severe conjunctivitis, ocular surface erosion, pseudomembrane, and onychopathy were positively correlated with all three factors in the chronic stage. Table 5 shows the result of the association between acute and chronic factors.

**Table 5:** Associations between acute and chronic findings.

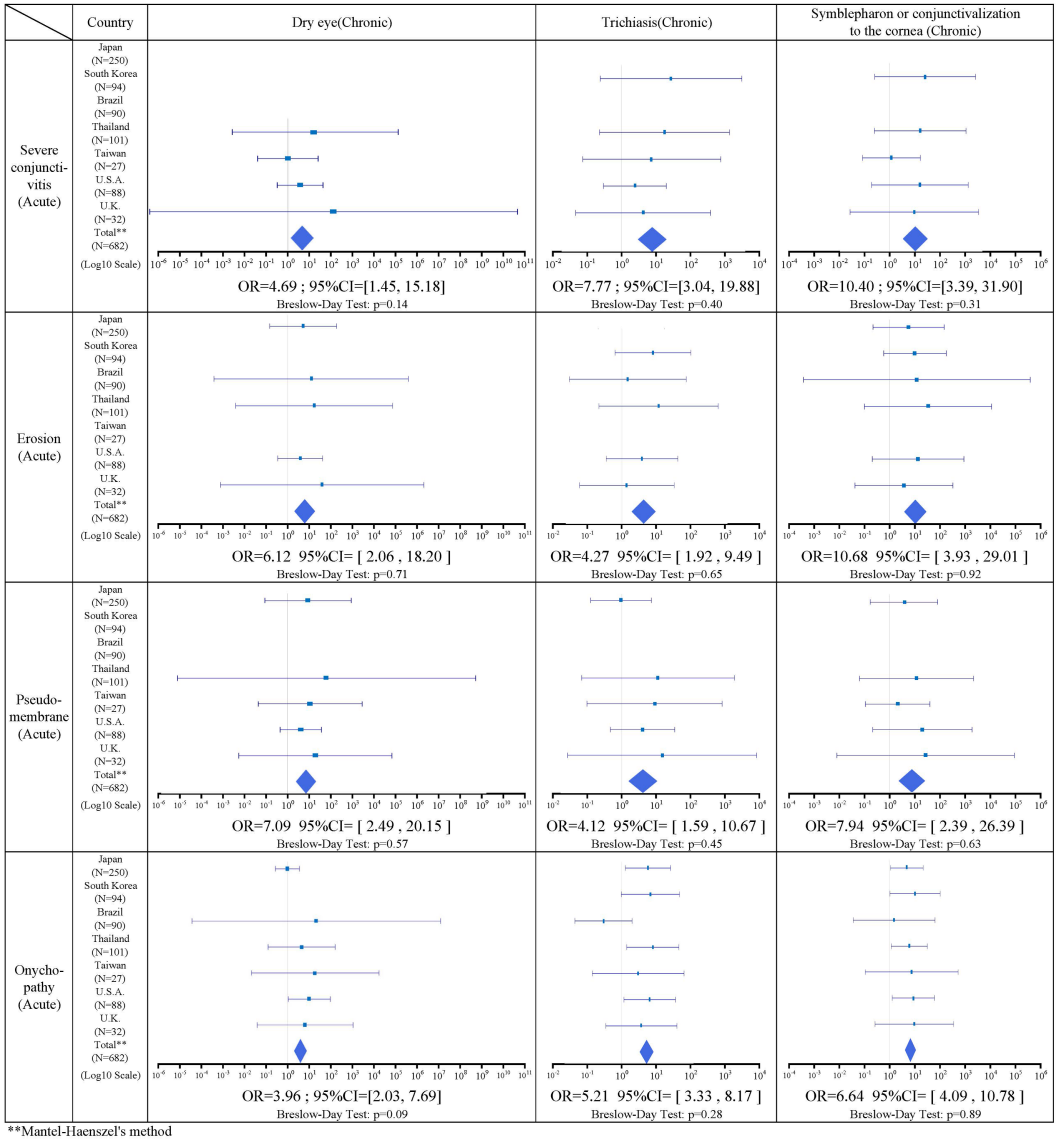

## Discussion

In all, data from 682 patients were obtained (Figure 1) from seven countries (Japan, Korea, Brazil, Thailand, Taiwan, United States, and United Kingdom; representative cases are shown in Supplementary Figure S1). There were 623 patients (91.3%) providing data on severe conjunctivitis with ocular surface erosion and/or pseudomembrane in the acute stage or with symblepharon or conjunctivalization of the cornea in the chronic stage. Of the patients with SOC, there was data on cold medication ingestion in 462 patients of which 296 (64.1%) were taking cold medications before the onset of SJS/TEN. Similarly, there was data on pre-onset use of antibiotics in 467 patients, of whom 153 (32.8%) were taking antibiotics before the onset of SJS/TEN. Anticonvulsant usage data were available for 467 patients, of whom 54 (11.6%) were taking anticonvulsants before the onset of SJS/TEN.

Regarding the association of medications with acute and chronic findings, ingestion of cold medications was significantly positively correlated with trichiasis (IOR = 1.73) and symblepharon or conjunctivalization of the cornea (IOR = 1.93) in the chronic stage. Dry eye was not statistically significantly associated, but showed a positive correlation trend (IOR = 1.49), although it was not significantly associated. The association between cold medicines and findings in the acute stage such as severe conjunctivitis, ocular surface erosion, and pseudomembrane were not significantly correlated. However, this study revealed that cold medicine-associated SJS/TEN is significantly more likely to result in ocular sequelae such as trichiasis and symblepharon or conjunctivalization of the cornea in the chronic stage of SJS/TEN. Therefore, those who take cold medications and develop SJS/TEN should be even more closely monitored for ocular findings because of the possibility of the high incidence of ocular sequela.

The use of antibiotics prior to disease onset was not correlated with dry eye, trichiasis, symblepharon, and/or conjunctivalization of the cornea in the chronic stage. The use of antibiotics before the onset of SJS/TEN was not significantly associated with acute or chronic findings. However, the association between antibiotics and all findings in the chronic stage tends to show a negative correlation. Ocular findings may be less likely to occur in SJS/TEN caused by antibiotics before the onset of SJS/TEN compared to those caused by cold medications. This result was consistent with the previous report by Sotozono et al. (1). Thus, cold medications might contribute more to the SOC of SJS/TEN, although antibiotics might be prescribed with cold medications for common cold symptoms in some countries.

Taking anticonvulsants before the onset of SJS/TEN was significantly negatively correlated with conjunctivitis (IOR = 0.33) and with oral mucosal findings (IOR = 0.21) in the acute stage. A significant negative correlation was also found between anticonvulsants and trichiasis in the chronic-stage findings (IOR = 0.41). Ocular findings may be less likely to occur in SJS/TEN caused by anticonvulsants before the onset of SJS/TEN compared to cold medications. This result was consistent with a previous report by Ma et al. (24).

Interestingly, the onset with anticonvulsant drugs tended to be about 2 weeks after medication (33), whereas the onset with cold medicine tended to be shorter, within 1 week in our study, as we previously reported (23, 34). The different duration between medication and onset might suggest that their mechanisms of the onset of SJS/TEN might be quite different.

Of the 643 patients with data for the presence or absence of common cold symptoms such as sore throat, fatigue, and slight fever, 437 (68.0%) had common cold symptoms before the onset of SJS/TEN. This indicates that common cold symptoms often precede the onset of SJS/TEN with SOC. Regarding the association of common cold symptoms with acute and chronic findings, common cold symptoms prior to the onset of SJS/TEN were significantly positively correlated with acute conjunctivitis (IOR = 3.23), and ocular surface erosions (IOR = 3.15). In addition, common cold symptoms before the onset of SJS/TEN were significantly positively correlated with trichiasis (IOR = 1.80) and symblepharon or conjunctivalization of the cornea (IOR = 1.58) in the chronic stage. Previously, Ueta et al. suspected that SJS/TEN might be caused when people with an unusual genetic predisposition took cold remedies for infections such as viruses. In this study, of 437 patients with confirmed common cold symptoms, 278 (63.6%) were taking common cold remedies, suggesting that both cold remedies, as well as microbial infections, may be involved in the pathogenesis of SJS/TEN (34–37).

Among the 682 patients, 423 were female (62.0%). This indicates that patients with SJS/TEN who presented with SOC tended to be female. The mean age of onset for the 672 patients for whom the age of onset was known was 29.1 years (SD 19.4 years). Regarding the association of age with acute and chronic findings, age more than 30 years was negatively associated with acute conjunctivitis (IOR = 0.42), ocular surface erosions (IOR = 0.43), pseudomembrane (IOR = 0.25) in the acute stage, and nail disorders (IOR = 0.61), trichiasis (IOR = 0.58), and symblepharon or conjunctivalization of the cornea (IOR = 0.50) in the chronic stage. This indicates that the age of onset of SJS/TEN of less than 30 years is more likely to result in SOC in the acute and chronic stages of SJS/TEN. Thus, children who develop SJS/TEN should be continuously monitored for ocular findings not only in the acute stage but also in the chronic stage, because of the possibility of high incidence of SOC.

Based on combined ophthalmology collaborative data from seven countries, acute findings of severe conjunctivitis were observed in 92.3% (383/415), ocular surface erosions in 86.1% (273/317), pseudomembranes in 86.2% (163/189), onychopathy in 77.5% (505/652), alopecia in 59.6% (212/356), and vesiculobullous lesion of the oral cavity and lips were present in 98.8% (559/566). Chronic ocular sequelae included dry eye in 90.6% (465/513), trichiasis in 73.2% (447/611), and symblepharon or conjunctivalization to the cornea in 74.9% (466/622). Regarding the association of acute stage findings with chronic stage findings, severe conjunctivitis in the acute stage was significantly positively correlated with dry eye (IOR = 4.69), trichiasis (IOR = 7.77), and symblepharon or conjunctivalization of the cornea (IOR = 10.4). Ocular surface erosions were significantly positively correlated with dry eye (IOR = 6.12), trichiasis (IOR = 4.27), and symblepharon or conjunctivalization to the cornea (IOR = 10.7). Pseudomembrane had a significant positive correlation with dry eye (IOR = 7.09), trichiasis (IOR = 4.12), and symblepharon or conjunctivalization to the cornea (IOR = 7.94).

These findings indicate that acute ocular findings such as severe conjunctivitis with ocular surface erosion and pseudomembrane in the acute stage are likely to cause ocular sequelae in the chronic stage. Thus, the SJS/TEN patients with SOC in the acute stage should be continuously monitored for ocular findings in their chronic stage, due to the high incidence of ocular sequelae.

Onychopathy was significantly positively correlated with dry eye (IOR = 3.96), trichiasis (IOR = 5.21), and symblepharon or conjunctivalization of the cornea (IOR = 6.64). Onychopathy in the acute stage was, therefore, likely to be associated with ocular sequelae in the chronic stage.

There are several limitations to our study. First, in many hospitals, acute ocular findings are not recorded. Of the 682 patients, only 170 (24.9%) records reflected the presence or absence of the full range of acute findings we sought in the study. For many of the patients in the study, an ophthalmologist was not called on in the acute stage. Another limitation is that paracetamol (acetaminophen) is available as an over-the-counter drug in the UK, the US, and Thailand, and it may not be reflected as a causative agent in some of the clinical reports.

Other confounding factors such as management in the acute stage and chronic stage were not taken into account in the analysis. The treatments in the acute stage such as steroid pulse therapy and amniotic membrane transplantation might affect the chronic ocular findings of the SJS/TEN patients (38). Previous studies have also shown that interventions such as lid-margin mucous membrane grafting and scleral contact lenses can significantly alter the natural course of the disease and prevent corneal complications in the chronic stage (39, 40). Thus, further investigations are needed including the effects of treatments in the future.

Moreover, in this study, we did not include the findings of lid-related disorders of SJS/TEN with SOC. Further investigations including them are also needed in the future.

In summary, this study assimilates worldwide data from patients with ocular SJS/TEN and shows that patients with SOC in the acute stage are more likely to have ocular sequelae in the chronic stage. It has been reported that ocular sequelae are less likely to occur if pulsed intravenous corticosteroid is administered within 4 days of onset (41), or amniotic membrane if placed within 6 days of onset (38). Early treatment in the acute stage is, therefore, critical for preventing SOC in the chronic stage of SJS/TEN. Importantly, in this large international series, cold medicines were strongly associated with SJS/TEN with SOC. Because patients take cold medicines for common cold symptoms, it is possible that viral or microbial infections, with or without the subsequent use of cold medicine, may contribute to the pathophysiology of SJS/TEN and ensuing SOC.

## Data availability statement

The original contributions presented in the study are included in the article/Supplementary material, further inquiries can be directed to the corresponding authors.

## Ethics statement

The studies involving human participants were reviewed and approved by KPUM. Written informed consent to participate in this study was provided by the participants’ legal guardian/next of kin.

## Author contributions

MU planned the study design. MU, CS, MK, TW, PJ, HS, SR, DM, KY, VP, CB, SA, KS, C-KJ, JG, JC, and SK were involved in the data collection. MU, CI, MN, and ST were involved in the data interpretation and data analysis. MU, CI, MN, ST, HS, CB, and JC wrote the manuscript. All authors critically revised the report, commented on drafts of the manuscript, and approved the final report.

## Conflict of interest

The authors declare that the research was conducted in the absence of any commercial or financial relationships that could be construed as a potential conflict of interest.

## Publisher’s note

All claims expressed in this article are solely those of the authors and do not necessarily represent those of their affiliated organizations, or those of the publisher, the editors and the reviewers. Any product that may be evaluated in this article, or claim that may be made by its manufacturer, is not guaranteed or endorsed by the publisher.
