# Supplementary material for: Severe ocular complications of SJS/TEN and associations among pre-onset, acute, and chronic factors: a report from the international ophthalmology collaborative group
Source: Front Med (Lausanne). 2023 Jun 22;10:1189140. doi: 10.3389/fmed.2023.1189140 (PMC10325566; doi:10.3389/fmed.2023.1189140)
Supplement: Supplementary file 1 [file Data_Sheet_1.PDF]

Supplementary Table1-A: The associations between taking anticonvulsants before the onset and findings in the acute stage

The association between Taking Anticonvulsants before the onset and Severe conjunctivitis at the acute stage

Country	Conjunctivitis (+) / Anticonvulsants (+)	Conjunctivitis (+) / Anticonvulsants (-)	OR	95%CI
Japan (N=250)	8/8(100.0%)	57/57(100.0%)	0.15 *	[0.01 , 2.95] *
South Korea (N=94)	3/4(75.0%)	54/65(83.1%)	0.61	[0.07 , 5.60]
Brazil (N=90)	24/24(100.0%)	61/61(100.0%)	0.40 *	[0.01 , 10.77] *
Thailand (N=101)	4/5(80.0%)	52/53(98.1%)	0.08	[0.01 , 0.61]
Taiwan (N=27)	2/4(50.0%)	17/17(100.0%)	0.03 *	[0.01 , 0.12] *
U.S.A. (N=88)	2/2(100.0%)	22/28(78.6%)	1.44 *	[0.05 , 39.38] *
U.K. (N=32)	2/2(100.0%)	6/7(85.7%)	1.15 *	[0.03 , 42.48] *
Total (N=682)	45/49(91.8%)	269/288(93.4%)	0.33**	[0.12 , 0.94]

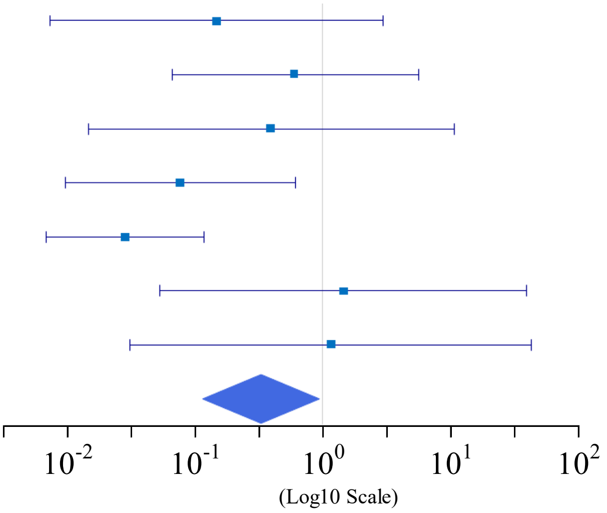

*Woolf Correction

**Mantel-Haenszel's method

Breslow-Day Test: p=0.46

The association between Taking Anticonvulsants before the onset and Vesiculobullous lesion of oral cavity and lips at the acute stage

Country	Oral disorders (+) / Anticonvulsants (+)	Oral disorders (+) / Anticonvulsants (-)	OR	95%CI
Japan (N=250)	14/14(100.0%)	148/149(99.3%)	0.29 *	[0.02 , 5.44] *
South Korea (N=94)	5/5(100.0%)	65/65(100.0%)	0.08 *	[0.00 , 1.54] *
Brazil (N=90)	24/24(100.0%)	60/60(100.0%)	0.40 *	[0.01 , 11.03] *
Thailand (N=101)	4/5(80.0%)	56/56(100.0%)	0.03 *	[0.00 , 0.14] *
Taiwan (N=27)	4/4(100.0%)	18/18(100.0%)	0.24 *	[0.01 , 5.72] *
U.S.A. (N=88)	3/3(100.0%)	32/32(100.0%)	0.11 *	[0.01 , 2.07] *
U.K. (N=32)	2/2(100.0%)	7/8(87.5%)	1.00 *	[0.03 , 33.32] *
Total (N=682)	56/57(98.2%)	386/388(99.5%)	0.21**	[0.06 , 0.80]

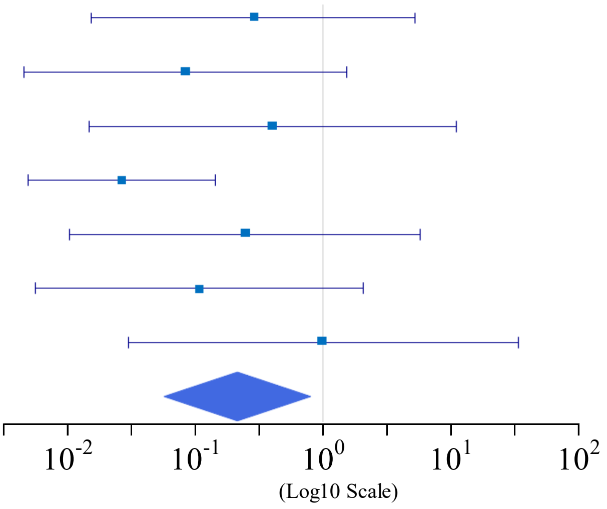

*Woolf Correction

**Mantel-Haenszel's method

Breslow-Day Test: p=0.78

Supplementary Table1-B: The associations between taking anticonvulsants before the onset and ocular findings in the chronic stage

The association between Taking Anticonvulsants before the onset and Trichiasis at the chronic stage

Country	Trichiasis (+) / Anticonvulsants (+)	Trichiasis (+) / Anticonvulsants (-)	OR	95%CI
Japan (N=250)	9/13(69.2%)	136/170(80.0%)	0.56	[0.18 , 1.75]
South Korea (N=94)	2/4(50.0%)	34/48(70.8%)	0.41	[0.07 , 2.29]
Brazil (N=90)	11/21(52.4%)	40/53(75.5%)	0.36	[0.15 , 0.83]
Thailand (N=101)	2/5(40.0%)	48/62(77.4%)	0.19	[0.05 , 0.74]
Taiwan (N=27)	2/4(50.0%)	14/17(82.4%)	0.21	[0.04 , 1.17]
U.S.A. (N=88)	1/3(33.3%)	24/38(63.2%)	0.29	[0.05 , 1.78]
U.K. (N=32)	1/2(50.0%)	1/7(14.3%)	6.00	[0.02 , 2146.86]
Total (N=682)	28/52(53.8%)	297/395(75.2%)	0.41**	[0.22 , 0.76]

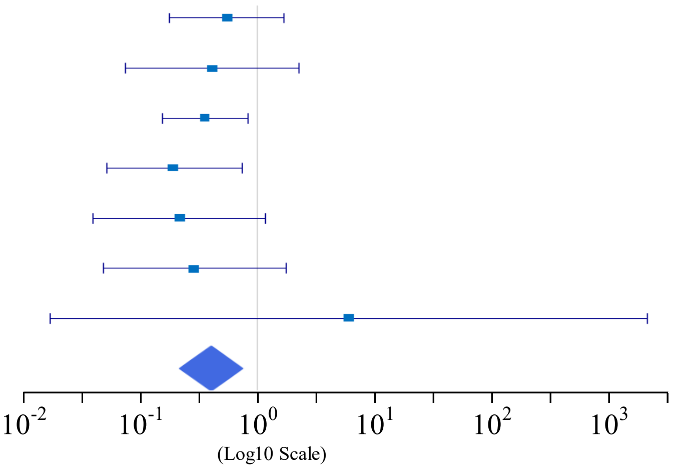

*Woolf Correction

Breslow-Day Test: p=0.61

**Mantel-Haenszel's method

Supplementary Table2-A: The associations between age on the onset and findings in the acute stage

The association between Age at the onset and Severe conjunctivitis at the acute stage

Country	Conjunctivitis (+) / Age>=30 (+)	Conjunctivitis (+) / Age>=30 (-)	OR	95%CI
Japan (N=250)	42/42(100.0%)	30/30(100.0%)	1.39 *	[0.02 , 104.51] *
South Korea (N=94)	38/47(80.9%)	25/30(83.3%)	0.84	[0.27 , 2.66]
Brazil (N=90)	22/22(100.0%)	66/66(100.0%)	0.34 *	[0.01 , 8.51] *
Thailand (N=101)	39/44(88.6%)	40/41(97.6%)	0.20	[0.05 , 0.70]
Taiwan (N=27)	7/10(70.0%)	14/15(93.3%)	0.17	[0.04 , 0.76]
U.S.A. (N=88)	15/20(75.0%)	22/24(91.7%)	0.27	[0.08 , 0.94]
U.K. (N=32)	10/11(90.9%)	10/10(100.0%)	0.33 *	[0.03 , 3.56] *
Total (N=682)	173/196(88.3%)	207/216(95.8%)	0.42 **	[0.20 , 0.91]

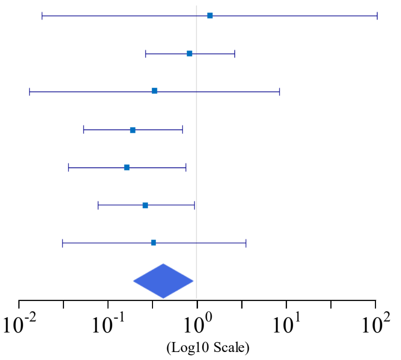

*Woolf Correction Breslow-Day Test: p=0.80

**Mantel-Haenszel's method

The association between Age at the onset and Erosion of ocular surface epithelium at the acute stage

Country	Erosion (+) / Age>=30 (+)	Erosion (+) / Age>=30 (-)	OR	95%CI
Japan (N=250)	23/28(82.1%)	19/19(100.0%)	0.11 *	[0.03 , 0.41] *
South Korea (N=94)	32/47(68.1%)	17/24(70.8%)	0.88	[0.31 , 2.47]
Brazil (N=90)	10/10(100.0%)	15/16(93.8%)	2.03 *	[0.05 , 84.32] *
Thailand (N=101)	39/44(88.6%)	33/33(100.0%)	0.11 *	[0.03 , 0.39] *
Taiwan (N=27)	9/10(90.0%)	16/16(100.0%)	0.19 *	[0.02 , 1.54] *
U.S.A. (N=88)	15/21(71.4%)	25/27(92.6%)	0.20	[0.07 , 0.61]
U.K. (N=32)	10/11(90.9%)	8/9(88.9%)	1.25	[0.06 , 27.72]
Total (N=682)	138/171(80.7%)	133/144(92.4%)	0.43 **	[0.21 , 0.87]

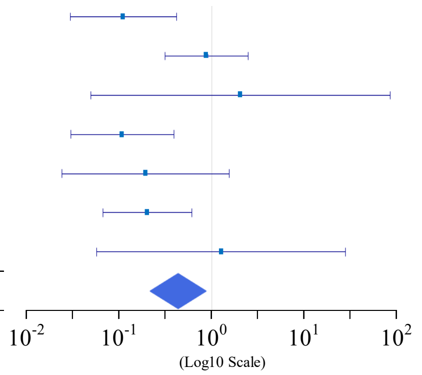

*Woolf Correction Breslow-Day Test: p=0.40

**Mantel-Haenszel's method

The association between Age at the onset and Pseudomembrane with conjunctivitis at the acute stage

Country	Pseudomembrane (+) / Age>=30 (+)	Pseudomembrane (+) / Age>=30 (-)	OR	95%CI
Japan (N=250)	24/28(85.7%)	18/18(100.0%)	0.15 *	[0.03 , 0.65] *
South Korea (N=94)	-	-	-	-
Brazil (N=90)	-	-	-	-
Thailand (N=101)	39/42(92.9%)	28/28(100.0%)	0.20 *	[0.04 , 1.05] *
Taiwan (N=27)	6/10(60.0%)	8/9(88.9%)	0.19	[0.04 , 0.85]
U.S.A. (N=88)	11/18(61.1%)	20/25(80.0%)	0.39	[0.13 , 1.16]
U.K. (N=32)	3/5(60.0%)	5/5(100.0%)	0.13 *	[0.02 , 0.79] *
Total (N=682)	83/103(80.6%)	79/85(92.9%)	0.25 **	[0.09 , 0.65]

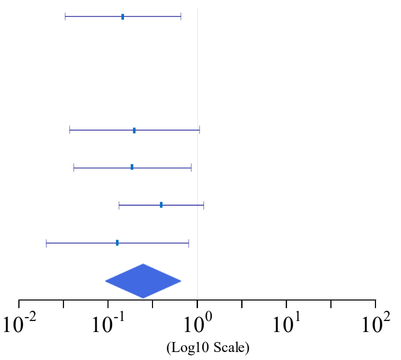

*Woolf Correction Breslow-Day Test: p=0.94

**Mantel-Haenszel's method

The association between Age at the onset and Onychopathy at the acute stage

Country	Onychopathy (+) / Age>=30 (+)	Onychopathy (+) / Age>=30 (-)	OR	95%CI
Japan (N=250)	86/99(86.9%)	109/124(87.9%)	0.91	[0.42 , 1.98]
South Korea (N=94)	33/58(56.9%)	18/36(50.0%)	1.32	[0.54 , 3.23]
Brazil (N=90)	20/22(90.9%)	64/66(97.0%)	0.31	[0.06 , 1.59]
Thailand (N=101)	24/47(51.1%)	42/51(82.4%)	0.22	[0.12 , 0.42]
Taiwan (N=27)	8/11(72.7%)	15/16(93.8%)	0.18	[0.04 , 0.81]
U.S.A. (N=88)	23/35(65.7%)	39/53(73.6%)	0.69	[0.29 , 1.61]
U.K. (N=32)	6/12(50.0%)	10/13(76.9%)	0.30	[0.08 , 1.06]
Total (N=682)	200/284(70.4%)	297/359(82.7%)	0.61 **	[0.41 , 0.90]

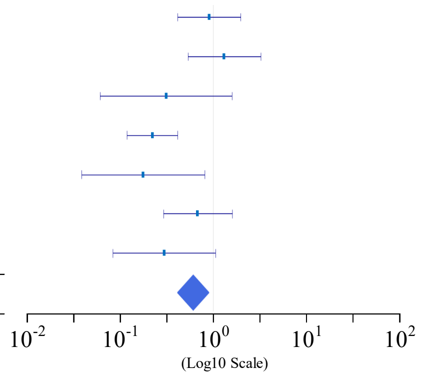

*Woolf Correction Breslow-Day Test: p=0.08

**Mantel-Haenszel's method

Supplementary Table2-B: The associations between age on the onset and ocular findings in the chronic stage

The association between Age at the onset and Trichiasis at the chronic stage

Country	Trichiasis (+) / Age>=30 (+)	Trichiasis (+) / Age>=30 (-)	OR	95%CI
Japan (N=250)	79/102(77.5%)	103/126(81.7%)	0.77	[0.42 , 1.41]
South Korea (N=94)	28/42(66.7%)	17/22(77.3%)	0.59	[0.21 , 1.63]
Brazil (N=90)	10/15(66.7%)	43/61(70.5%)	0.84	[0.26 , 2.68]
Thailand (N=101)	29/45(64.4%)	42/52(80.8%)	0.43	[0.20 , 0.91]
Taiwan (N=27)	3/9(33.3%)	14/15(93.3%)	0.04	[0.01 , 0.10]
U.S.A. (N=88)	21/34(61.8%)	36/49(73.5%)	0.58	[0.26 , 1.33]
U.K. (N=32)	6/14(42.9%)	8/15(53.3%)	0.66	[0.18 , 2.46]
Total (N=682)	176/261(67.4%)	263/340(77.4%)	0.58**	[0.40 , 0.84]

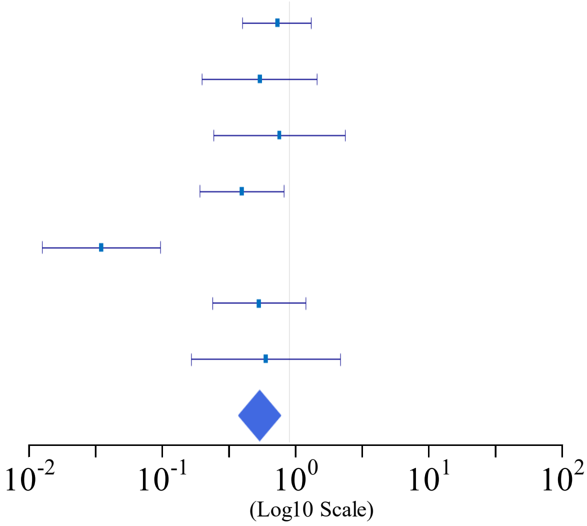

*Woolf Correction Breslow-Day Test: p=0.24

**Mantel-Haenszel's method

The association between Age at the onset and Symblepharon or conjunctivalization to the cornea at the chronic stage

Country	Symblepharon (+) / Age>=30 (+)	Symblepharon (+) / Age>=30 (-)	OR	95%CI
Japan (N=250)	84/108(77.8%)	114/131(87.0%)	0.52	[0.29 , 0.93]
South Korea (N=94)	28/42(66.7%)	17/22(77.3%)	0.59	[0.21 , 1.63]
Brazil (N=90)	15/15(100.0%)	55/61(90.2%)	3.63 *	[0.14 , 97.07] *
Thailand (N=101)	30/45(66.7%)	43/52(82.7%)	0.42	[0.20 , 0.90]
Taiwan (N=27)	0/9(0.0%)	9/15(60.0%)	0.04 *	[0.01 , 0.10] *
U.S.A. (N=88)	14/33(42.4%)	26/50(52.0%)	0.68	[0.30 , 1.52]
U.K. (N=32)	9/14(64.3%)	13/15(86.7%)	0.28	[0.07 , 1.03]
Total (N=682)	180/266(67.7%)	277/346(80.1%)	0.50**	[0.34 , 0.75]

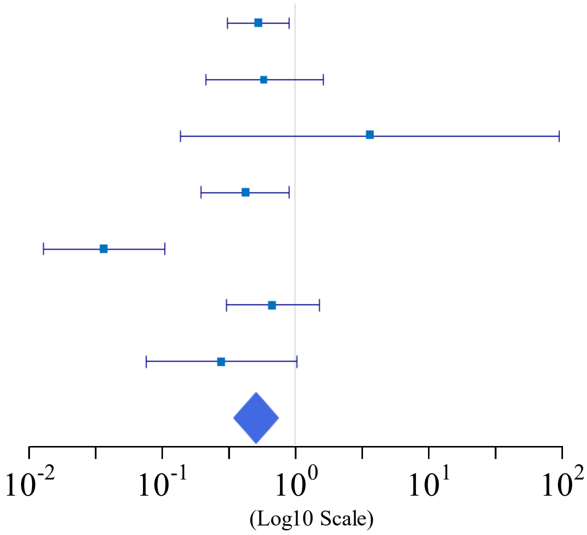

*Woolf Correction Breslow-Day Test: p=0.27

**Mantel-Haenszel's method
