# Supplementary figures and images for: Severe ocular complications of SJS/TEN and associations among pre-onset, acute, and chronic factors: a report from the international ophthalmology collaborative group
Source: Front Med (Lausanne). 2023 Jun 22;10:1189140. doi: 10.3389/fmed.2023.1189140 (PMC10325566; doi:10.3389/fmed.2023.1189140)

Supplementary Figure 1

JAPAN

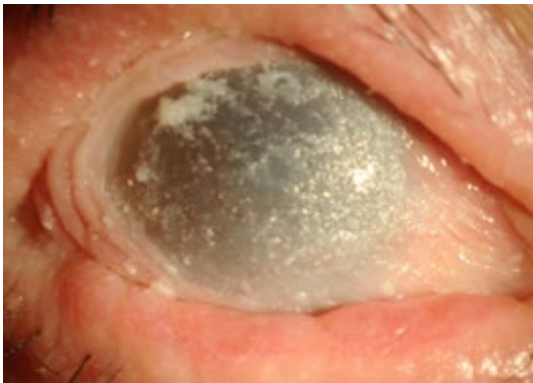

KOREA

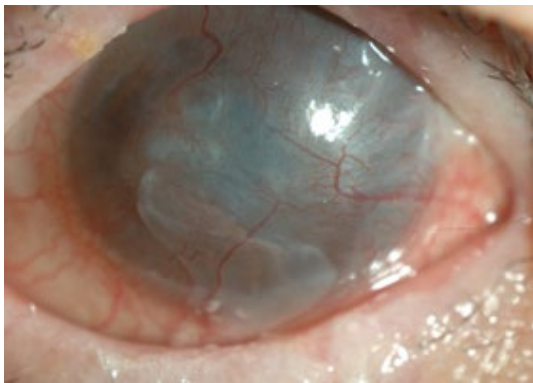

BRAZIL

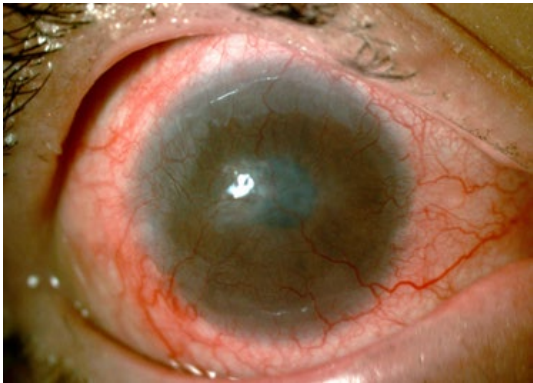

THAILAND

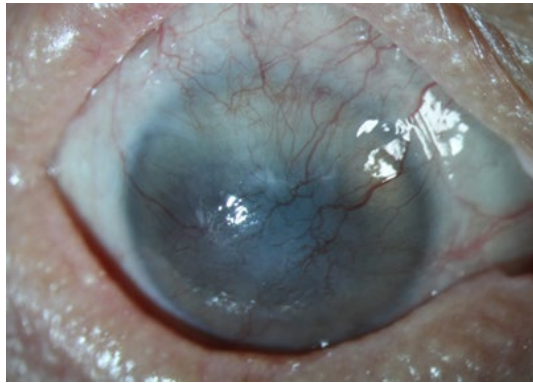

TAIWAN

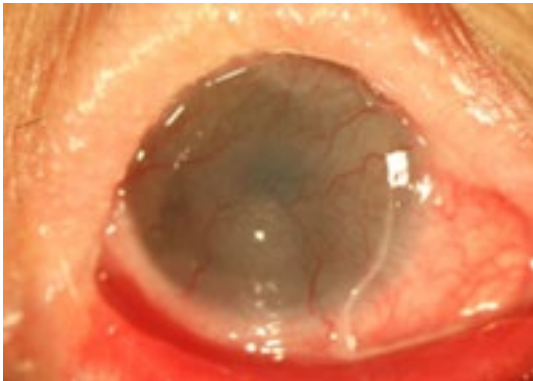

USA

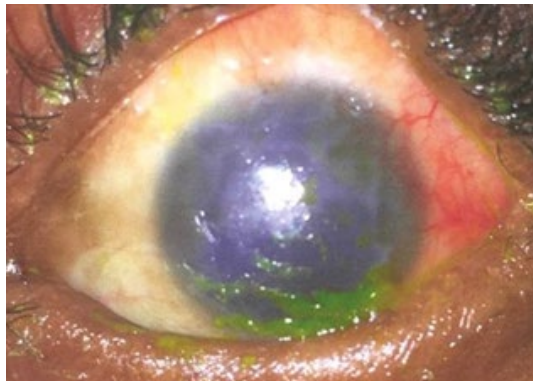

UK

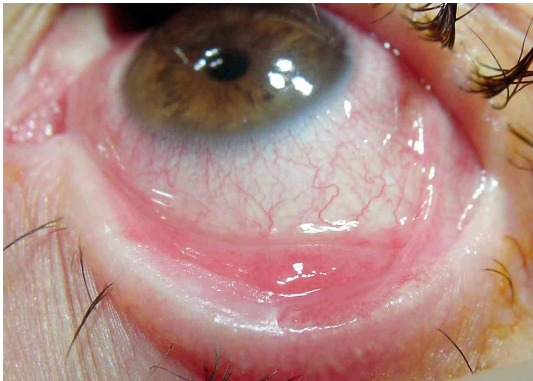

Supplement: Supplementary file 2 [file Data_Sheet_2.PDF]
